# Supplementary material for: Are carbon emissions trading and green financial instruments synergistic? -Comprehensive quantitative research based on content analysis
Source: PLoS One. 2024 Mar 7;19(3):e0298601. doi: 10.1371/journal.pone.0298601 (PMC10919601; doi:10.1371/journal.pone.0298601)
Supplement: S1 Data — (ZIP) [file pone.0298601.s001.zip › Data/地级市Z_F工作报告.zip!/╜¡╬≈/╛░╡┬╒≥╩╨/└┤╫╘╣1⁄2╓┌║┼í╛┬φ┐╦╩2╛▌═°í┐_2010.docx]

各位代表：

现在，我代表市人民政府向大会作政府工作报告，请予审议，并请各位市政协委员和列席会议的同志提出意见。

一、2009年的工作回顾

过去的一年，在省委、省政府和市委的正确领导下，全市上下坚持以邓小平理论和“三个代表”重要思想为指导，深入学习实践科学发展观，全面落实党的十七大、十七届三中、四中全会和胡锦涛总书记视察江西“三保一弘扬”重要讲话精神，以大项目推动大发展，以高投入拉动快增长，以促改革创新增活力，以重民生实现大稳定，较好地克服了国际金融危机的不利影响，全市经济社会实现了平稳持续较快发展。

2009年，全市实现生产总值364.03亿元，增长13.8%；完成财政总收入35.55亿元，增长25.1%，其中一般预算收入24.33亿元，增长31.4%；全社会固定资产投资331亿元，增长43.7%；社会消费品零售总额118.54亿元，增长19.4%；城镇居民人均可支配收入14996元，增长10.4%；农民人均纯收入5705元，增长8.6%；人口自然增长率7.82‰。

项目建设快速推进。始终坚持“发展抓项目”的理念，全市115项重点工程项目完成投资123亿元。重大基础设施项目建设取得新成效，电厂“上大压小”项目正式开工，完成投资15.1亿元；九景衢铁路、皖赣铁路复线改造项目建议书获国家发改委批复；浯溪口水利枢纽项目前期工作进展顺利；煤矿棚户区改造项目落实国家投资计划1.47亿元，首批7148万元已下达；德兴至南昌高速公路（乐平段）开工建设；天然气、景德镇机场等新建、扩建项目建设进展顺利。成功列入国家第二批资源枯竭型城市，已争取到位中央财政财力性转移支付资金2.04亿元。产业项目快速推进，光伏产业基地单晶硅项目、长虹景德镇机电产业基地项目、景德镇汽车零部件产业基地项目、深圳华烨LED产业项目、莱特陶瓷以及乐华、金意陶二期工程等一大批产业项目相继建成投产。

开放型经济蓬勃发展。围绕产业抓招商，围绕招商兴产业。美国NEA和百奥维达基金收购立信药业全部股权，南京中电、浙江金龙化工、沈阳同联集团、台湾正翰科技、台湾兴勤电子、日本爱和陶、福建汉索夫、中国节能投资公司、中国电力投资集团、上海威富尔新能源科技、湖北致远科技集团公司等一大批企业落户我市，开放型经济实现高速增长。全年新增市外3000万元以上项目153个，实际进资165.08亿元，同比增长70.49%，其中：5000万元以上工业项目进资93.72亿元，增长28.23%；全市新审批外资项目17个，实际利用外资1.01亿美元，增长14.9%；外贸出口逆势进位，出口总量4.81亿美元，增长116.9%。2009景德镇国际陶瓷博览会成功举办，参展企业600余家，瓷博会期间内贸成交总额6.23亿元，外贸成交总额1.07亿美元。我市首次获得全省开放型经济发展综合奖。

农村经济稳步增长。粮食播种面积超额完成省政府下达的目标，达到138.78万亩；粮食总产连续六年实现增长，再创历史新高，达到56.75万吨，增长4.13%。农业产业化经营进程加快，规模以上农业产业化龙头企业实现销售收入31亿元，增长8.8%。新农村建设和农村清洁工程试点工作扎实推进，202个省级新农村建设点共投入资金1.26亿元，161个建设点初步形成了“一村一品”的产业格局。“造地增粮富民工程”成效明显。“一大四小”工作有效开展，完成造林面积19.45万亩。农业基础设施建设成效显著，农业综合开发完成总投入7172.5万元，争取省以上水利项目资金1.8亿元，增长52.5%，完成42座病险水库除险加固任务，完成水土流失治理5.3万亩。

工业化进程不断加快。把“保企业”放在经济工作的突出位置，促进了工业经济的健康发展。全市完成规模以上工业增加值145.19亿元，增长21.5%；新增规模以上工业企业83户，全市总计达到414户；工业经济效益综合指数达到195.38%，比上年提高20.57个百分点。园区发展势头强劲，三大园区规模以上工业实现主营业务收入217.65亿元，占全市规模以上工业主营业务收入的45.8%。陶瓷产业突破四个大关，产值总量突破100亿元大关，达到100.3亿元；出口突破1亿美元大关，达到1.08亿美元；上缴税收突破1亿元大关，达到1.02亿元；陶瓷规模企业数量突破100家大关，达到102家。

城乡面貌继续改善。以城市“五创”为龙头，全面推进城市规划、建设和管理。科学编制各类规划，城区详规覆盖率达90%。重点工程建设扎实推进，新五中建成并投入使用；体育场、妇幼保健院、光明电影院主体工程封顶；市广场人行天桥投入使用；建成六个景观戏亭；完成了人民广场周边和“一江两岸”建筑立面改造；完成了浮桥西路、中华北路延伸南段工程等道路改造和十八渡人行浮桥工程；全市公厕免费向市民开放。民窑遗址博物馆和御窑遗址保护工程建设进展顺利。加大土地调控力度，保障了重点项目建设用地。大力开展无证私房清理整顿，有效遏制了违章建筑的滋生和蔓延。城市创建工作取得新成效，通过了全国城市公共文明指数测评，再次荣获江西省文明城市和江西省卫生城市称号。《景德镇市加快小城镇建设实施方案》颁布实施，搭建重点小城镇建设项目融资平台，全市16个乡镇共33个项目列为第一批融资建设项目。36个乡镇全面完成了农村垃圾处理场的建设任务。

各项改革卓有成效。国企改革从资金筹措、债务化解、人员安置、产业发展等关键问题入手，创新思路，积极推进，实现重大突破。全面完成列入全省国有工业企业改革调度的48户企业改革，职工身份置换率达到99.75%。财政改革稳步实施，市本级全额拨款单位国库集中支付改革全面启动，基本实现财政资金国库集中支付的全覆盖；非税收入电子化票据改革全面完成；公务卡制度改革工作正式启动。金融支持经济发展力度进一步增强，全市人民币各项存款余额319亿元，增长27.8%，净增69亿元，各项贷款余额179亿元，增长30.5%，净增41.8亿元，增量和增幅创近十年历史最高水平。金融体制改革不断深化，市城信社和市农信社组建商业银行工作取得实质性进展；市、县两级政府投融资平台基本建设完成，全年实现融资近15亿元；机关事业单位国有资产统一经营管理工作全面完成。启动了医药卫生体制改革。农村土地承包合同和农村土地承包经营权到户率均达到98.6%以上，林权制度配套改革继续推进。

文化旅游业发展势头强劲。全年接待旅游人数1078万人次，增长18.7%，旅游总收入53亿元，增长21.22%。陶瓷历史文化博览区、御窑厂、高岭-瑶里风景区、浮梁古县衙、洪岩风景区等重点景区景点功能建设不断完善。中国瓷园成功创建国家AAAA级景区，海畅集团和陶瓷股份公司成功列为江西省工业旅游示范点，古窑·民俗博览区镇窑复烧成功，并申报吉尼斯纪录。民窑博物馆、祥集弄民居和三闾庙古码头等老城区旅游项目建设快速推进。景德镇陶瓷文化创意产业基地获省批复并授牌。“辉煌60年-景德镇陶瓷成就成果展”在北京和台北成功举办，近二十万中外人士参观；《大瓷商》等一批影视作品在中央电视台等媒体播出，进一步扩大了瓷都景德镇的影响。

民生工程扎实推进。坚持以人为本，关注民生，财政支出进一步向民生工程倾斜，2009年市本级安排民生工程配套资金8600多万元，比上年新增1700万元。全市城镇新增就业2.35万人，城镇就业率达96.5%；“4050”人员新增就业3568人，零就业家庭安置率100%，农村劳动力转移培训3万人，城镇职业培训2.77万人。社会保障水平不断完善和提高，全年征缴养老保险基金4.84亿元；失业保险基金2959.5万元。新型农村合作医疗参合率97.13%；实施“光明·微笑”工程，免费施行白内障手术4501例，唇腭裂手术213例。城市和农村低保标准由每人每月200元、90元分别提高到215元、100元；城镇“三无人员”供养标准提高到每人每月300元。开工建设南河、昌明经济适用住房小区共8万平方米，新建廉租住房2820套，面积14.1万平方米；中心城区廉租住房租赁补贴保障家庭累计达6903户，发放廉租住房补贴1442万元。为农村困难群众新建、重建住房176户。完成黄乐线、景湖线、田乐线、吴乐秧线和景瑶线等五条省道改造95公里。完成东三线等18条线路大中小修135公里。行政村通水泥（油）路100%，新开工建设渡改桥项目25个。完成20户以上自然村“村村通”广播电视工程126个。新增解决农村安全饮水人口23.07万人，农村沼气新建户数4233户。

社会事业协调发展。科技创新取得新成效，我市被列为科技部全国五个创新型城市试点规划项目城市之一。争取国家科技支撑计划项目1个和国家创新基金项目6个，申报国家重点新产品和火炬计划项目6个。陶瓷学院、602所、昌飞公司被科技部授予国家级“国际科技合作基地”称号。我市荣获省科技进步奖4个、省自然科学奖1个。教育事业加快发展，初中辍学率控制在2.2%以内，高中阶段毛入学率达74.49%；新增3所省重点中学和5所省重点建设中学。京剧现代戏“水点桃花”剧本荣获第23届田汉戏剧奖剧本二等奖。《景德镇传统手工制瓷技艺》通过文化部审核。第三次文物普查工作继续推进，普查覆盖率100%。我市文化工作获得“全国文化系统先进集体”荣誉称号。甲型H1N1流感得到有效控制。市第二医院、第三医院和妇幼保健院荣获三甲医院称号。乐平市、浮梁县被评为全省农村卫生工作先进县。生态环境综合治理进一步强化，完成了年度减排任务。全面开展药品安全专项整治，确保了食品药品安全。成功举办全市第十一届运动会，我市运动员在第十一届全国运动会上夺得三枚金牌。景德镇老年大学被授予“全国先进老年大学”称号。切实抓好“安全生产年”各项工作，安全生产形势进一步稳定好转。着力化解各类社会矛盾，圆满完成各个敏感节点的维稳任务；刑事案件下降，治安状况好转率为全省第一；信访数量下降，赴京赴省访总量为全省最少之一。“五五”普法取得阶段性成效，办理法律援助案件905件，法律援助事项10200起。人口出生率为11.5‰，计划生育率为87.26%，出生婴儿性别比100：107。国防后备力量建设进一步加强，驻市部队和民兵预备役官兵积极参建，市国动委指挥中心正式建成并启动运行。统计调查服务功能进一步增强。同时，人事、编制、国家安全、供销、气象、水文、工商、新闻出版、民族宗教、双拥、老龄、妇女儿童、残联、人防、档案、地方志、保险、通迅、无线电管理等各项事业也取得了新的进步。

自身建设不断加强。主动接受人大及其常委会的法律监督、政协及其常委会的民主监督。及时办理人大代表意见建议86件，办复率100%，办结率56%；政协提案128件，办复率100%，办结率56%。认真开展机关效能年建设活动，建立健全了以“首问责任制”、“限时办结制”、“责任追究制”等三项制度为核心的机关效能建设制度。进一步加大了行政审批制度改革力度，行政许可项目和投资项目审批事项办理时间压缩率分别为45.5%和43.3%。市公共资源交易中心顺利建成运行，为重大产业项目开辟“绿色通道”。全市43个乡镇建立了便民服务中心，在294个行政村设立了398个为民服务代办点。深入推进政务公开，发布了办事依据、办事条件、办事程序，设计并公开了运行流程图。我市机关效能建设荣获全省11个设区市第一名。

各位代表，过去的一年，面对国际金融危机的严重冲击，面对严峻复杂的经济形势，全市上下努力化危为机，攻坚破难，实现了我市经济和社会事业的全面进步。这些成绩的取得，是省委、省政府和市委坚强领导的结果，是市人大、市政协大力支持、有力监督的结果，是全市人民团结奋斗、辛勤劳动的结果。在此，我代表市人民政府，向全市广大工人、农民、知识分子、干部和离退休老同志，向各民主党派、工商联、无党派人士和社会团体，向驻市解放军、武警官兵和公安干警，向所有关心、支持瓷都发展的海内外友好人士，表示衷心的感谢，并致以崇高的敬意！

在看到成绩的同时，我们也清醒地看到经济社会生活中还存在不少困难与问题。主要是经济总量仍然偏小，总体发展水平还不高；财政收支压力较大，金融债务还比较重；就业还不够充分，部分群众生活仍比较困难，影响社会和谐稳定的因素还不少；经济发展环境和行政效率仍有待进一步改善和提高等。对于这些困难和问题，我们将高度重视，并通过发展在今后的工作中努力克服和解决。

二、2010年工作任务和主要措施

2010年是实施“十一五”规划的最后一年，做好今年经济社会发展工作，对进一步有效应对金融危机，巩固经济回升基础，促进全市经济跨越发展，为“十二五”规划启动实施创造良好条件至关重要。

2010年市政府工作的总体要求是：坚持以科学发展观为指导，全面贯彻落实党的十七大、十七届三中、四中全会和中央经济工作会议精神，抓发展，调结构，促转变，惠民生，以积极融入鄱阳湖生态经济区建设、加快城市转型和建设国家科技创新型城市为契机，以工业化为核心，以大开放为主战略，以项目建设为载体，以加快新型城镇化为抓手，继续增加投入、做大总量，促进我市在鄱阳湖生态经济区的跨越式发展。

2010年全市国民经济和社会发展的主要目标是：全市生产总值增长13%；财政总收入、地方财政一般预算收入增长13%；全社会固定资产投资增长30%；社会消费品零售总额增长16%；外贸出口增长10%；实际利用外资增长10%；实际引进内资增长20%；规模以上工业增加值增长20%；城镇居民人均可支配收入增长8%；农民人均纯收入增长8%；人口自然增长率控制在8‰以内；万元生产总值能耗下降5.7%。

（一）扎实推进项目建设，增强经济发展实力和后劲

投资是经济增长的重要推动力。必须把抓项目、扩投资作为经济发展的核心，全力以赴抓好项目建设。坚持“两动”，实现“三增”，即坚持项目带动、投资拉动；实现经济发展速度增快、总量增大、后劲增强。全年全社会固定资产投资力争增长30%，达到430亿元。

积极争取重大基础设施项目。准确把握国家政策导向，结合全国、全省“十二五”规划编制和鄱阳湖生态经济区建设，精心组织好事关全市经济发展大局的在建、续建和争取报批的重大项目建设。景德镇电厂第一台机组年内建成发电；景德镇机场新候机楼建成投入使用；天然气实现通气。积极争取浯溪口项目尽快批复，并列入2010年开工计划。开工建设九景衢铁路、皖赣线复线改造和祁（门）浮（梁）高速公路项目，复工建设绕城公路南环段，加快德昌高速（乐平段）建设。继续争取棚户区改造、御窑保护、景德镇国际陶瓷文化创意服务平台、高技术陶瓷产业基地等项目政策与资金支持。加快500千伏景德镇变电站建设进度，开工建设220千伏南山变电站、鱼山开关站。

加快推进重大产业项目建设。着力抓好景德半导体多晶硅项目、长虹机电产业项目、嘉和汽配城项目、现代能源锂离子动力电池项目、威富尔新能源项目、密胜家电产业基地项目和金龙化工草甘膦等重大产业项目建设，提升产业支撑能力。推进在谈的香港坤达薄壁不锈钢管、广州昊勤铝电解电容器、深圳广合昌LED芯片等项目，尽快签约、进资、落户。抓好已进资的合众锦川陶瓷、新耀华药业、明月建陶等项目开工、建设和投产。

千方百计提高项目投资效益。严格抓好项目资金的使用和管理，高度重视项目的开工、投产，按进度完成重大工程投资量和实物工作量。国债资金项目做到开工建设100%，地方配套资金落实100%，对检查中发现的问题整改到位100%。加快项目建设进度，提高项目建设质量，努力将项目尽快转化为现实生产力，使项目更好更快地发挥应有的经济效益、社会效益。

（二）积极融入鄱阳湖生态经济区建设，加快资源转型发展

抓住鄱阳湖生态经济区建设上升为国家战略的有利时机，利用好国家资源枯竭型城市转型的政策支持，积极融入，主动对接，着力调整产业结构，转变发展方式，实现科学发展、绿色崛起、进位赶超。

做好融入鄱阳湖生态经济区建设规划。融入鄱阳湖生态经济区建设既是一项重大战略，又是一项系统工程。作为鄱阳湖生态经济区东部的一个中心城市，我市要主动融入大局，提升发展思路，按照高起点、高标准、高水平的要求，结合“十二五”规划编制工作，突出产业特色、生态特色和文化旅游特色，科学做好景德镇在鄱阳湖生态经济区的城市发展定位、产业布局和项目规划。坚持规划先行、协同推进、优势互补，做产业，成系统，可持续。

优化产业发展布局。坚持以工业化为核心，围绕光伏、LED半导体照明、航空制造、新材料高技术陶瓷、生物和新医药、清洁汽车及动力电池、现代农业和有机食品、文化及创意产业等八大战略性新兴产业，突出工业项目，狠抓工业投入。全市规模以上工业增加值增长20%，达到178亿元。以中国直升机研究所和昌飞公司为主体，建设航空直升机产业园，发展壮大航空制造产业；抓住昌汽兼并重组机遇，全力以赴支持汽车产业做大做强；以乐平精细化工园和焦化集团为载体，延长产业链，加快精细化工产业聚集；以德宇集团和乐平蔬菜集团等企业为重点，发展现代农业和有机食品产业；大力培育以多晶硅、单晶硅、太阳能电池为主体的新材料、新能源产业。加快长虹机电产业基地建设，压缩机产量力争达到1800万台，冰箱50万台。

加快陶瓷产业转型升级。充分把握好国家资源枯竭型城市转型的机遇，找准接续替代产业，重点发展高科技陶瓷和陶瓷文化创意产业，实现传统陶瓷产业的转型升级。依托国家日用及建筑陶瓷工程技术研究中心和陶瓷工业园区，大力发展新材料和高技术陶瓷，形成高科技含量、高附加值、高市场占有率的高技术陶瓷产业集群。2010年，全市陶瓷工业总产值达到150亿元；陶瓷出口1.2亿美元。大力发展陶瓷文化创意产业，以产业支撑为平台，重点扶持陶瓷文化创意产业龙头企业，积极吸引中小创意企业及个人创业者，打造国际性陶瓷文化艺术交流、创作、展示和交易平台，做大陶瓷艺术创意新兴产业规模。要选好转型项目，严格申报审批，完善政策配套，推进项目建设，实现转型升级。

加强生态环境建设和保护。大力推进造林绿化“一大四小”工程，以通道绿化为重点，抓好国道、省道、高速公路两旁及可视范围的绿化，努力打造九景高速公路、206国道、景瑶观光线、乐德线等四条通道绿化精品；结合新农村建设，进一步开展村庄绿化；抓好城区和市高新区、乐平工业园区、陶瓷工业园区等“四区”造林绿化。大力推进节水型社会建设，重点抓好工农业节水示范项目。强化节能减排，推进永虹活性炭厂节能技术改造、华电高压电器有限公司能量系统优化工程等一批项目，积极开展低碳经济试点和机关节能工作。加大对重点行业环境污染的整治，强化工业企业污染物排放总量控制和排污许可制度执行情况的监督检查。确保城市污水处理厂正常运转，实现达标排放。2010年，全市化学需氧量排放量控制在2.17万吨以内，二氧化硫排放量控制在4.01万吨以内，努力完成省政府下达的“十一五”期间万元GDP能耗降低22.6%的目标。

（三）加速新型城镇化进程，提升城镇发展质量和水平

推进新型城镇化是扩大内需、调整结构的重要抓手，是推动经济跨越发展的重要引擎，是实现经济转型的重要突破口和关键环节。要以景德镇中心城区和乐平、浮梁县城建设为重点，促进城市和小城镇协调发展，不断增强区域中心城市和中心城镇的聚集力和辐射带动力。

科学编制城市规划。坚持依法规划城市，依法建设城市，依法管理城市，依法保护城市。坚持规划的统一性、连续性、权威性。完成《景德镇市城市总体规划（2008-2030年）》修编工作。围绕城市建设工作重点，加快近期建设规划、各类专项规划和近期建设地段控制性详规等30项规划的编制工作，力争控规覆盖率达到100%。突出山水园林和生态文化特色，注重城市设计，做好老城区和重点片区建设和保护规划。同时，认真做好县城和重点乡镇的规划。

加快城市重点工程建设。以新型城镇化建设为抓手，掀起新一轮城市大建设、大发展新高潮。以休闲广场至中国陶瓷博物馆为轴线，重点建设昌南大道沿线城市新区；改造一江两岸，昌江东岸保留传统特色，昌江西岸凸显现代气息；以窑砖色为主色调，打造“镇派”城市风格，把景德镇建设成山水、文化、生态特色城市。建设“一个中心”，实施“五个一”工程，全面推进城市项目建设。即围绕休闲广场，着力构建包括珠山路、浙江路、广场南路等商业街以及沃尔玛、金鼎百货、新天地、商贸大厦等商业楼盘在内的城市中心商业圈；打造“一条江”，即一江两岸绿化亮化美化；“一座桥”，即开通白鹭大桥；“一条路”，即贯通景东大道片区；“一批馆”，即建设完工景德镇陶瓷馆、体育场、光明电影院等；“一组园”，即建设陶艺文化墙主题公园、森林公园、云门公园、松涛公园等。以棚户区改造为契机，完成戴家弄、斗富弄、人民公园周边改造工程，启动韭菜园、西瓜洲老城区改造项目，加快改造老城区排水主干管道，开工建设市垃圾处理场，实施供水管网改造三期工程。

创新城市管理机制。以城市“五创”为抓手，建立健全科学、系统、完整的城市管理体系。严厉打击违法用地和违章建筑，实现城市建设有序发展。加快农贸市场标准化升级改造，加强规范化管理。做好土地储备、出让和融资工作，提高土地市场运作效益。积极开展城市创建工作，通过中央文明办组织的对我市城市文明指数的第二次测评；通过国家卫生城市省级考核和国家环保模范城市省级预验收；争创江西省生态园林城市和全国未成年人思想道德建设工作先进城市。

推进小城镇建设。把城镇化建设与新农村建设结合起来，在做大做美中心城市的同时，以工业化和农业产业化促进城镇化进程，加强以乐平、浮梁县城和建制镇为重点的城镇化建设。科学规划小城镇布局，以示范镇建设为引导，重点扶持工业重镇、商贸集镇建设。以中心城区为核心，放射状沿高速公路、国省道建设好乡（镇）政府所在地的小城镇，发挥小城镇的集聚辐射带动功能。把解决符合条件的农业转移人口逐步在城镇就业和落户，作为推进城镇化的重要任务，在提供公共服务、放宽城镇户籍准入、实现养老保险转移接续等方面加大推进力度。2010年，全市城镇化率提高1个百分点，达到57%。

（四）夯实“三农”发展基础，扩大内需增长空间

围绕“稳粮保供给、增收惠民生、改革促统筹、强基增后劲”的工作要求，确保粮食生产不滑坡，确保农民收入不徘徊，确保农村发展好势头不逆转，为全市经济跨越发展提供更加有力的支撑。

加快发展现代农业。围绕农业增效和农民增收，稳定粮食种植面积，落实种粮直补和农机具购置补贴政策，抓好农业关键技术推广应用，千方百计提高粮食单产和种粮效益。2010年，全市农业总产值增长6%，粮食播种面积138万亩，粮食总产稳定在55万吨以上。大力推进农业产业化，调整优化农业结构，继续实施农业产业化“121”工程和“双十双百双千”工程建设，加快发展壮大蔬菜、茶叶、养殖等主导产业，鼓励企业发展绿色食品和有机食品。进一步做大品牌农业规模，扶持龙头企业做大做强，扩大“得雨活茶”、“浮瑶仙芝”、“绿乐蔬菜”、“赣森茶油”等优势农产品的品牌影响力。2010年，力争培育规模以上龙头企业超过80家，全市农业产业化经营销售收入突破40亿元，增长10%。

加强农业基础建设。围绕改善农业生产条件，继续实施好国家农业综合开发项目，进一步加强农田水利基础设施建设。重点抓好昌江河、乐安河的治理工程和玉田水库扩建工程立项工作，抓好乐平润泉供水工程建设。加大病险水库除险加固力度，完成列入国家规划内20座病险水库和市级47座水库应急除险加固任务，完成全市小型水库安全状况普查。

推进新农村建设。按照“生产发展、生活宽裕、乡风文明、村容整洁、管理民主”的要求，抓重点，攻难点，重特色，成规模，由点至线成面，完成200个左右新农村试点村建设。加大“一村一品”建设力度，力争65%以上的建设点形成“一村一品”产业。着力抓好以“三清六改四普及”和“三绿一处理”为主要内容的村庄清洁绿化工程建设，抓好农村垃圾无害化处理，切实改善农村生活环境。

（五）坚持改革开放，增强经济发展活力和动力

实现我市经济跨越发展，必须以改革开放破解难题、转变机制、激发活力、提升水平。要坚定不移地实施大开放主战略，推进体制机制创新，深化各项改革，不断增强经济发展活力和动力。

稳步推进各项改革。完善国有企业改革，积极稳妥做好破产清算、国有资产监管等后续工作。尽快完成11户企业的政策性关闭破产，做好省属下放企业改革、企业社区移交等工作。引进战略投资者，加快陶瓷股份公司、焦化工业集团战略重组。稳妥推进农村各项改革，稳定和完善农村基本经营制度，继续推进林权制度配套和农村综合改革。深化财税改革，加强预算监督和管理，市本级部门预算编制进一步科学化、精细化，县（市、区）全面开展部门预算试点，进一步深化集中支付、非税收入管理、政府采购改革，全面推行公务卡，建立财政投入绩效评价制度。积极推进政府机构改革，不断深化医药卫生体制改革。完善机关事业单位国有资产统一划转和经营管理工作。深化投融资体制改革，支持焦化工业集团、世龙实业公司等重点企业上市融资；完成市城信社和市农信社组建商业银行工作；争取组建我市第一家村镇银行；鼓励发展融资担保主体，缓解融资难问题。加强政银企协作，进一步发挥金融在现代经济发展中的核心作用，鼓励金融机构扩大信贷投放，增强企业直接融资能力，力争全年实现融资30亿元。进一步放宽民间资本的市场准入条件，鼓励和引导民间资本投资基础设施、新兴产业和社会公共事业，促进民营经济健康快速发展。

坚持大开放主战略。主攻园区平台建设，围绕在全省园区发展中争先进位的目标，突出招商引企，突出项目投入，突出企业投产，突出高效服务。大力开展招商引资，紧紧盯住重点地区、重点产业、重点企业、重点客商，瞄准全球500强和国内500强企业上门招商，瞄准中央企业和其他重点国企定向招商，瞄准与我市产业关联度高、带动能力强的行业龙头企业开展产业招商。不断扩大外贸出口，加快出口型企业基地建设，组织落实好各项外贸出口扶持政策，加大对重点出口企业的扶持力度，培植新的出口增长点。

（六）弘扬陶瓷历史文化，加快文化旅游产业发展

注重发挥我市陶瓷历史文化资源优势，重点是打好一张牌（瓷都牌），唱好一台戏（瓷博会），打造一条线（精品旅游线路），促进陶瓷文化资源优势向旅游产业优势转化，全面提升瓷都文化旅游的整体水平。2010年，全市接待国内外游客1240万人次，旅游总收入62亿元。

完善景区景点建设。强化陶瓷文化博览区、高岭-瑶里景区、古县衙等重点景区功能，建设陶瓷文化博览区和瑶里游客服务中心。完善锦绣昌南-中国瓷园和得雨生态园景区的旅游基础配套设施。积极推进洪岩景区二期工程。加快御窑等景区景点保护建设步伐。启动旅游商品研发集散中心项目建设。注重城市老街区的保护、开发和利用，精心打造御窑、建国、艺术、民窑老城区陶瓷文化旅游线。加快旅游公路建设，改善景区景点交通环境。

加快旅游资源整合。推进以陶瓷文化为龙头、特色品牌为依托的旅游资源整合和旅游发展合作。积极支持旅游企业通过投资、收购、兼并、参股、资产重组等方式，扩大产业规模，提升竞争力。重点开发以陶瓷文化、自然山水、购物观光、休闲度假等为主体的多层次、复合型精品旅游线路。

积极拓展旅游市场。以上海世博会和广州亚运会为契机，加强与周边城市的旅游合作。强化旅游宣传，更新、完善旅游宣传图册，配合机场航班航线加密，加大旅游促销力度，带动长三角、京津塘和珠三角地区的客源市场。全面落实旅游营销奖励政策。继续打造瓷博会、瓷文化旅游节、茶文化旅游节等会展节庆品牌和御窑、民窑、镇窑等旅游产品，塑造“千年瓷都、艺术之城”的国际旅游形象。

（七）坚持以人为本，着力保障和改善民生

坚持公共财政向困难群众倾斜、向农村倾斜、向基层倾斜、向公共事业倾斜，重点解决最困难群众最迫切需要解决的突出问题。2010年，市本级财政将安排1亿多元用于民生工程，重点支持就业、社保、教育、医疗和住房等方面。

扩大就业促进创业。实施积极的就业政策，完善就业服务体系，千方百计增加就业，鼓励创业。加大对大学生就业创业扶持力度。积极帮助困难群众就业，零就业家庭就业安置率100%。新增转移农村劳动力1.8万人。劳动力转移培训1.14万人，其中创业培训2300人。

完善社会保障制度。扩大社会保险覆盖面，加强社保基金征缴。城镇基本养老保险参保缴费21.47万人，基金征缴总额5.79亿元。新型农村社会养老保险参保7万人，被征地农民参加养老保险2.9万人，失业保险基金征缴2600万元。加大医疗保障力度，逐步建立健全覆盖城乡的基本医疗卫生制度。城镇职工和居民基本医疗保险参保51.2万人，城镇职工工伤保险参保23.8万人，农民工参加工伤保险6.1万人。城镇居民基本医疗保险覆盖率达到100%。完善新型农村合作医疗制度，统筹基金使用率85%以上。做好城镇女职工生育保险工作。免费提供社区公共卫生服务卡360万元。同时，健全救助体系，提高救助标准。农村五保户集中供养率80%以上。提高精简退职老职工救济金水平，其中城市达到225元、农村达到185元。

加强保障性住房建设。进一步推进经济适用住房和廉租住房建设，并为农村困难群众提供住房帮助。为农村困难群众新建、重建住房196户，为农村困难群众维修住房107户。

提高农村群众生产生活水平。推进扶贫开发，移民扶贫搬迁180人，整村推进重点扶贫村12个。农村使用沼气新增3000户。积极开展送戏下乡，为农村放映电影7766场。建设农民体育健身场所28个。解决1278个自然村垃圾处理问题。

认真为民办好十件实事。1、扩大就业。新增城镇就业2.31万人，城镇就业率94.5%以上，解决“4050人员”就业0.17万人。2、提高居民低保标准和补助水平。城市低保和农村低保月人均补差水平分别由155元和65元提高到180元和75元；城镇集中供养“三无”人员的标准从每人每月300元提高到每人每月400元，农村五保户集中供养和分散供养标准分别由每人每年1860元和1260元提高到1920元和1320元。3、继续实施农村渡改桥工程。年内完成渡改桥28座。完成206国道和景白线省道46公里大中修，完成135公里农村公路改造。4、加快国企改革社区移交，完成移交社区改水改电工程。5、完善市政基础设施。完成市第三期城市供水管网改造。贯通白鹭大桥和景东大道，开通和延伸2条公交线路。6、方便群众出行。完成全市人行道及960个停车泊位修缮工程，对银曙路、太白园、新枫路、昌西路、解放路、太平巷、麻石弄、黎明路、中山北路延伸等9处路段大修改造。7、完善市民休闲设施。完成陶瓷文化墙主题公园、森林公园、云门公园和松涛公园建设。8、完善城市功能。完成人民来访接待中心、体育中心体育场、光明电影院、妇幼保健院、市中医院综合大楼、民窑博物馆建设，完善御窑遗址博物馆续建工程。9、搞好保障性住房建设。全市改造棚户区22.72万平方米，市中心城区完成1000套廉租住房建设。10、继续实施“光明·微笑”工程，实施治安防控体系二期工程建设。

（八）统筹经济社会，推进各项事业协调发展

加快发展社会事业，是以人为本，建设和谐社会的必然要求，也是扩大消费、拉动内需的重要内容。必须加大工作力度，注重协调各方面利益关系，统筹经济与社会发展，加快推进和谐瓷都建设。

推进“科教兴市”战略。以建设国家创新型城市为抓手，建立健全科技创新机制体制，搭建科技创新平台，打造一个充满生机和活力的创新型城市。进一步推进省部共建景德镇国家陶瓷科技城，抓好已经列入国家、省科技计划重大项目的实施和验收，扎实做好国家知识产权试点城市工作。坚持教育优先发展战略，促进教育均衡发展，以提高教育质量为中心，推进素质教育；巩固提高“两基”成果，切实提高农村幼儿教育和高中教育普及率，狠抓农村控辍保学工作，义务教育阶段免学杂费和免费提供教科书学生数均达到19.5万人，资助普通高中家庭经济困难学生2700人，资助中等职业教育家庭经济困难学生6300人，资助考入大学家庭经济困难学生800人。改善学校办学条件，调整城乡教育布局，化解城区大班额问题，解决好农民工子女就学困难。推进高中新课程实验。全力抓好职教招生、培训工作，对接工业园区，实行校企合作，扩大中等职业教育办学规模，加紧规划建设市职业教育示范校。协调发展特殊教育、学前教育和老年教育。

大力发展文化卫生体育事业。促进文化事业繁荣发展，完善公共文化服务体系建设，实施文化惠民工程，不断提高公共文化的覆盖面和服务水平。进一步繁荣文艺创作和演出。完成20户以上自然村“村村通”广播电视工程30个。全面启动有线电视数字化整体转换。加强重大疾病预防控制，全面提高突发公共卫生事件应急处置水平。村卫生室（所）达标23个，实行国家基本药物制度的乡镇中心卫生院12个。完善覆盖城乡居民的食品药品安全保障体系，深入开展食品药品安全专项整治。认真贯彻《全民健身条例》，发展群众体育运动，进一步提高竞技体育水平，积极申办2014年第十四届省运会。加快多普勒气象雷达建设，提高对各类灾害性天气预报的时效性和准确率。继续稳定低生育水平，不断提高人口素质，综合治理出生人口性别比偏高问题。

推进和谐平安瓷都建设。发展高于一切，稳定压倒一切，安全稳定是最大的民生。全面加强应急管理工作，不断提高处置各类公共突发事件的能力和水平。建立健全“党委领导、政府监管、行业管理、企业负责、社会监督”的安全生产体系，坚决落实安全生产领导责任制和事故责任追究制。深化重点领域安全生产专项整治，坚决杜绝重特大事故，努力减少较大和一般事故，确保非法小煤窑继续实现“零开采、零死亡”。高度重视信访工作，多深入农村、社区和厂矿，了解基层群众诉求，把问题解决在萌芽状态。继续实施法律援助工程。深化法制宣传教育，全面完成“五五”普法规划。进一步加大严打整治工作力度，严厉打击涉黑、涉枪、涉恶、涉暴等严重暴力犯罪和抢劫、抢夺、盗窃等多发性侵财犯罪活动，强化社会治安集中整治，有效遏制各类违法犯罪活动，增强群众的安全感。大力推进国防后备力量和民兵预备役部队建设；进一步密切军政军民关系，争创全国双拥模范城“五连冠”。加强社区建设，增强服务功能，打造和谐社区。发展基层民主，构建充满活力的乡村治理机制。

（九）以行政效能建设为重点，加强政府自身建设

坚持科学执政、民主执政、依法执政，以“创业服务年”活动为抓手，努力建设“和谐、创新、务实、高效、自律”政府。

增强领导发展能力。坚持解放思想，更新观念，锐意进取，不断提高领导科学发展的能力。坚持用发展的思维和办法应对新情况，解决新问题，不断提高各级领导求发展、谋跨越的预见性、前瞻性和主动性。积极把握国家政策导向，进一步提高各级政府特别是各级领导干部抓项目、谋项目、上项目的工作水平，用抓项目的本领来真正体现抓经济工作的能力，用抓项目的业绩来衡量抓发展的成效。

提高服务发展水平。扎实开展“创业服务年活动”，不断提升创业服务水平。牢固树立“支持企业有责，成就企业有功”的理念，全力营造政府鼓励创业、社会支持创业、劳动者积极创业的浓厚氛围。进一步建立健全投资创业服务体系，积极为创业者提供项目开发、创业培训、开业指导、政策咨询、融资贷款、政策扶持、跟踪指导等服务，努力创建优质高效的政务和服务环境、宽松透明的市场和企业经营环境、优越的创新创业成长环境、公平正义的法制和社会环境。

积极推进依法行政。严格按照法定权限和程序行使职权、履行职责，努力提高政府工作的规范化、法制化水平。对重大政策、重大项目、重大投资的实施，坚持做到充分听取各方面意见，做到依法决策、科学决策和民主决策。自觉接受人大及其常委会的法律监督和政协的民主监督，认真听取各民主党派、工商联、无党派人士和人民团体的意见，接受新闻舆论和社会公众监督。推行政务公开，推进行政审批制度改革，深化“两集中、两到位”，提高政府工作透明度，确保权力正确行使。进一步强化市行政服务中心功能，规范和完善市公共资源交易中心运行机制，加快构筑全市统一的网上办事平台。

勤政廉政严格自律。以惩治和预防腐败为重点，以制约和监督权力为核心，以提高制度执行力为抓手，加强反腐倡廉制度建设。落实干部廉洁自律各项规定，扎实推进廉政“阳光工程”建设。强化对财政资金和政府投资工程的审计监督，深入开展治理建设工程等重点领域腐败问题。大力弘扬艰苦奋斗优良传统，进一步转变干部作风。健全厉行节约相关制度，严格控制公款消费、公费出国出境考察、购车，把有限的财力用在保增长、保民生、保稳定上。加强公务员廉政教育和管理，廉洁从政，勤政为民。

各位代表，2010年是“十一五”规划的最后一年，做好今年的各项工作，意义重大，使命光荣！让我们更加紧密地团结在以胡锦涛同志为总书记的党中央周围，高举中国特色社会主义伟大旗帜，深入贯彻落实科学发展观，在省委、省政府和市委的正确领导下，进一步解放思想，坚定信心，团结拼搏，排难前进，为实现瓷都在鄱阳湖生态经济区的跨越发展作出新的更大贡献！
